# Comparative Analysis of Total Laparoscopic Hysterectomy Versus Non-descent Vaginal Hysterectomy for Benign Uterine Pathologies in Women: A Systematic Review

**DOI:** 10.7759/cureus.62846

**Published:** 2024-06-21

**Authors:** Abhishek Sonkusare, Prachi Dixit

**Affiliations:** 1 Department of Obstetrics and Gynaecology, NKP Salve Institute of Medical Sciences and Research Centre, Nagpur, IND

**Keywords:** adenomyosis, fibroid uterus, salpingo-oophorectomy, benign uterine pathologies, non-descent vaginal hysterectomy, total laparoscopic hysterectomy

## Abstract

Hysterectomy, which can be conducted through abdominal or vaginal routes, is one of the most common gynecological procedures performed worldwide. When the patient is not able to undergo a vaginal hysterectomy due to contraindications involving a narrow pelvis or endometriosis and technical difficulties, laparoscopic removal of the uterus is the recommended method over abdominal hysterectomy. Additionally, the type of surgery depends on the expertise of the surgeon. Therefore, this systematic review aimed to evaluate different measures related to total laparoscopic (TLH) versus non-descent vaginal hysterectomy (NDVH) in women with benign uterine pathologies. ScienceDirect, PubMed, and Google Scholar databases were searched from 2019 to 2023 for a literature review using keywords including “Non-descent Vaginal Hysterectomy,” AND “Total Laparoscopic Hysterectomy,” AND “Benign Uterine Pathologies.” This systematic review includes five studies based on the selection criteria. The data were extracted and a quality assessment of the studies was performed. The review concluded that NDVH has an advantage over TLH as a scarless surgery performed in a very short period and with minimum blood loss with fewer complications and in terms of cost-effectiveness. However, the postoperative parameters and satisfaction with the TLH technique were better than the NDVH technique, but the procedure was much more time-consuming and needed laparoscopic surgical expertise. The duration of hospitalization in NDVH and TLH was nearly the same. Furthermore, both techniques could be employed for salpingo-oophorectomy or when there are adnexal masses and adhesions present; however, TLH may be the best course of action.

## Introduction and background

Hysterectomy is considered one of the most common gynecological procedures globally that can be performed either through the vaginal or the abdominal route. When a vaginal hysterectomy is not possible for a patient, a laparoscopic approach for uterus removal is a better option than an open abdominal hysterectomy (AH). In India, hysterectomy rates range from 4% to 6%, and the most common reason (90% of cases) is benign conditions [1,2]. Particularly, in rural areas, although AH is a more invasive procedure, it is still the most opted route for surgery mainly because of the following three reasons: (1) patients are not aware of the advantages of total laparoscopic hysterectomy (TLH); (2) the cost of surgery is lower when performed through the abdominal route; and (3) the majority of surgeons find it simple and easy to perform [3]. Although less invasive surgeries such as laparoscopy-assisted vaginal hysterectomy (LAVH), non-descent vaginal hysterectomy (NDVH), and TLH provide faster recovery times and cosmetic benefits compared to routine AH, they require more technical skills [3].

NDVH is considered a highly skilled, scarless, minimally invasive technique which is a highly preferable technique and can be performed safely for fibroid sizes larger than 12 weeks [4]. As NDVH is administered through a natural orifice, it is more efficient, quicker, and less expensive. NDVH performs better than TLH at remote hospitals with limited resources because it is less expensive, takes less time, can be completed with readily available equipment, and involves fewer surgical procedures [5]. In contrast to the traditional total AH, this surgical method results in less pain. Gynecologists are undoubtedly hesitant to use NDVH despite its demonstrated benefits because of its inability to conduct oophorectomy and technical challenges [6]. TLH leads to higher hospital costs but is gaining popularity because of its clear benefits, which include reduced patient morbidity, shorter hospital stays, and the ability to enable a direct view of the uterus and adnexa before any surgical dissection. However, it requires specialized laparoscopic equipment and updated infrastructure [6]. Before beginning TLH, it is crucial to have the right training and monitoring in place to reduce complications [7-9].

However, a Cochrane review and similar recommendations by the American College of Obstetricians and Gynecologists stated that NDVH should be preferred over AH wherever possible. Due to its more favorable adverse effect profile, laparoscopic procedures can be employed to avoid the drawbacks of a laparotomy in cases where a vaginal hysterectomy is not feasible [6,10,11]. Hence, the objective of this systematic review is to compare various measures associated with NDVH versus TLH in women with benign uterine diseases.

## Review

Methodology

This systematic review was performed following the Preferred Reporting Items for Systematic Reviews and Meta-Analyses (PRISMA) criteria [12].

Data Sources and Search Strategy

A systematic literature search was performed on electronic databases including ScienceDirect, PubMed, and Google Scholar databases from 2019 to 2023. The keywords utilized to perform the literature search and include relevant articles included “Total Laparoscopic Hysterectomy,” AND “Non-descent Vaginal Hysterectomy,” AND “Benign Uterine Pathologies.”

Study Screening and Selection

For screening, the inclusion criteria consisted of studies involving women in which a hysterectomy was performed for benign uterine pathologies either through the TLH or NDVH route, and a comparative analysis between both techniques was conducted. The review included cross-sectional studies, observational studies, randomized controlled trials, and retrospective observational studies conducted between 2019 and 2023. Finally, studies published in the English language with full-text availability were included. However, study designs that consisted of case reports, commentaries, guidelines, editorials, book chapters, and letters to editors; studies not reported in the English language; studies for which the full text was not available; and studies providing insufficient information related to the context were excluded.

The articles were evaluated by two reviewers independently to ascertain their suitability for inclusion in the review. First, to remove duplicates, titles and abstracts were screened. Second, the articles that were selected were screened again to remove articles not following the eligibility criteria. Finally, the selected articles were screened based on the full text to determine eligibility. Any discrepancies or disagreements among the reviewers were resolved through consensus and discussions.

Data Extraction

The data were extracted independently from the articles by the authors that included the first author along with the year of publication, study design, the sample size or number of patients involved, the age range of the patients, indication for hysterectomy, objective of the studies, methodological details, results derived, conclusion, and quality assessment. All extracted data were reviewed and combined.

Quality Assessment

The assessment of methodological quality for the included studies was conducted using the Mixed Methods Appraisal Tool (MMAT). This tool is commonly employed to evaluate studies that are qualitative, quantitative descriptive (cross-sectional), non-randomized, randomized controlled trials, and mixed methods [13,14]. The studies were graded as high, low, or moderate quality based on the various parameters described in the tool.

Data Synthesis

The critical narrative technique was employed to synthesize the results of the included studies. The narrative synthesis is described as the use of text, tables, and figures to summarize and validate study findings [13]. Higher methodological quality studies, incorporated study limitations, potential biases, and other factors were taken into consideration during the analysis of the findings, offering a critical perspective for the review. As relevant studies were limited in number, a meta-analysis approach or statistical synthesis was not suitable. Various research methodologies and outcome measures were considered in the included studies, leading to a significant degree of heterogeneity.

Results

**Figure 1:**
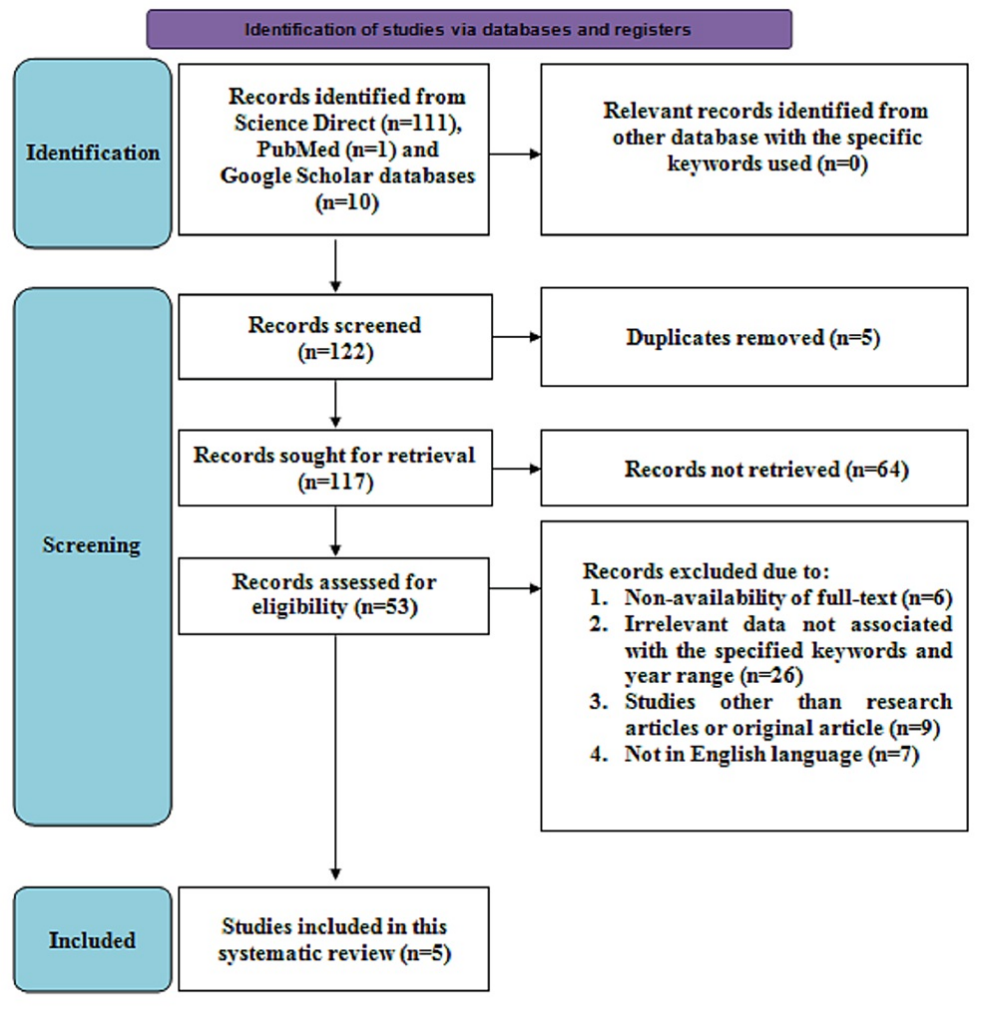
Search strategy based on the Preferred Reporting Items for Systematic Reviews and Meta-Analyses guidelines.

Figure 1 depicts the PRISMA search strategy flow diagram. Initially, 122 articles were screened consisting of 111 studies from the ScienceDirect database, one from the PubMed database, and 10 articles from the Google Scholar database. After removing the five duplicate articles, 117 articles remained and were evaluated for retrieval, of which 64 articles were not retrieved. Following this, 53 articles were screened for eligibility, of which 25 articles provided irrelevant data associated with the specified keywords, six articles reported non-availability of the full text, nine were studies other than research or original articles, and seven were not described in English language and were excluded. Hence, a total of five studies consisting of prospective comparative, analytical, and observational studies describing comparative outcomes from TLH and NDVH techniques for benign uterine pathologies were included in this systematic review.

A summary of the extracted data consisting of the first author and publication year, study design, sample size, the age range of the patients, and indication for hysterectomy from the five studies included is described in Table 1.

**Table 1:** Demographic parameters of the included studies. AUB/DUB = abnormal uterine bleeding/dysfunctional uterine bleeding

Author and year	Study design	Sample size	Age (years)	Indication for hysterectomy
Kanti et al. (2022) [2]	Prospective analytical study	145	30–60	Fibroid uterus, adenomyosis
Murali et al. (2019) [4]	Prospective comparative study	80	41–50	Fibroid uterus
Tondge et al. (2021) [6]	Prospective and comparative study	80	41–45	Fibroid uterus, adenomyotic uterus, and hyperplasia
Kansara et al. (2020) [15]	Retrospective comparative study	90	41–50	Adenomyotic uterus
Nath et al. (2022) [16]	Prospective observational study	100	32–62	AUB/DUB, uterine fibroids, adenomyosis, endometrial hyperplasia, endometriosis, and cervical in situ neoplasia

The objective, methodology, results, conclusions, and assessment of the quality of the included studies are demonstrated in Table 2.

**Table 2:** Comparative analysis between TLH and NDVH techniques. TLH = total laparoscopic hysterectomy; LAVH = laparoscopic-assisted vaginal hysterectomy; NDVH = non-descent vaginal hysterectomy; AH = abdominal hysterectomy; Hb = hemoglobin; PCV = packed cell volume

Author and year	Objective	Methodology	Results	Conclusion	Quality assessment
Kanti et al. (2022) [2]	To compare TLH, NDVH, and LAVH for the operative data and postoperative complications	Based on the admission into the hospital, 39 TLH cases, 60 NDVH cases, and 46 LAVH cases were included in the study. Blood loss, intra and postoperative complications, duration of surgery, and hospital stay were compared among the three groups during the procedure	In the TLH group, 126.79 ± 8.7 minutes was the mean operative time, in the LAVH group, it was 102.45 ± 10.53 minutes, and in the NDVH group, it was 54.67 ± 15.67 minutes, which was the shortest. In comparison to the LAVH (91.85 ± 10.66 mL) and the NDVH (59.50 ± 16.7 mL) groups, the TLH group showed the highest blood loss intraoperatively (111.025 ± 20.8 mL). Moreover, all three groups reported similar duration of hospitalization, with the TLH group experiencing more intra and postoperative complications	When there is an indication of salpingo-oophorectomy or when there are adhesions and adnexal masses present, TLH and LAVH may be better options. For more experienced surgeons, as NDVH is less invasive, deals with less blood loss, and is scarless, it may be the preferred approach	High
Murali et al. (2019) [4]	To determine which technique is superior for hysterectomy in a non-descent uterus by comparing the intra and postoperative complications of TLH versus NDVH	The patients were categorized into 40 cases each in NDVH and TLH. The two groups were compared based on the demographics, operating time, intraoperative blood loss, surgical indications, postoperative analgesic requirements, complications, and duration of hospitalization	The mean value for TLH was 120 minutes, whereas for NDVH it was 40 minutes related to operation time with statistically significant results. In the TLH and NDVH groups, the mean value was 120 versus 50 mL related to blood loss. No laparotomy conversions occurred in the NDVH group, while the TLH group showed three conversions. With statistically significant outcomes, both groups’ postoperative analgesic requirements, duration of hospitalization, and complications were found to be similar. Bilateral salpingo-oophorectomy by vaginal access was performed in six NDVH cases. Rectocele and cystocele correction was an additional benefit. Similarly, bilateral salpingo-oophorectomy was performed in six patients in the TLH group. The other common additional operation in the TLH group was adhesiolysis. In the TLH group, bladder injury and ureteric injury were observed in two cases and one case, respectively, which were transformed into laparotomy to manage complications. There were no intraoperative complications in the NDVH group. Both groups had the same analgesia needs and the same duration of hospitalization consisting of an average of three days. In the TLH group, one case of ileus was observed, whereas in the NDVH group, two cases of diarrhea and one case of pelvic abscess were observed postoperatively	Compared to TLH, NDVH is a less complicated and scarless surgical method	High
Tondge et al. (2021) [6]	To compare postoperative pain in patients who underwent TLH and NDVH, along with the blood loss, duration of the techniques, and complications	The study involved 40 patients in the NDVH group and 40 in the TLH group	With statistically significant results, the average operation time TLH was 112.5 minutes, and for NDVH, it was 55.2 minutes. The mean blood loss in NDVH was found to be lower (202.44 ± 29.92 mL) compared to the TLH group (206.88 ± 53.32 mL) but was not significant. The mean hospitalization days for TLH were 4.7 days, whereas for NDVH, there were 4.26 hospitalization days, with a significant difference. Urinary tract infection was the most frequent complication in the study for both TLH and NDVH, followed by pyrexia. Compared to TLH, there was a lower conversion rate from NDVH to laparotomy (total abdominal hysterectomy). With statistically significant results, the mean pain score in TLH was 4.42 ± 1.39, and in NDVH, it was 3.75 ± 1.01	When comparing the results and cost-effectiveness of the two surgical approaches, NDVH is determined to be superior to TLH.	High
Kansara et al. (2020) [15]	To determine which technique is superior for hysterectomy in a non-descent uterus by comparing the duration of the procedure, the drop in blood Hb level, and intra and postoperative problems between TLH and NDVH	The study involved 45 cases each in the TLH and NDVH groups. The two groups were compared for surgical indications, demographics, duration of surgery, intraoperative blood loss, analgesic requirements, complications, and hospital stay postoperatively. Patients with malignancies determined by D and C or Pap smear tests were not allowed to participate in the trial	In TLH, the surgeries were performed for approximately 80 minutes on average, while in NDVH, the surgeries were performed for 45 minutes. Considering intraoperative blood loss between the TLH and NDVH groups (80 versus 150 mL), there was a statistically significant difference. Similarly, compared to the NDVH group, the average duration of hospitalization for NDVH versus TLH was 3.4 days versus 2.5 days, respectively. In the TLH group, the requirement of blood transfusions postoperatively, and changes in the average Hb levels were much lower. The need for postoperative analgesia and complications following surgery were found to be similar in both groups	When patient satisfaction and postoperative parameters were compared, TLH might be superior to NDVH; nevertheless, it necessitates laparoscopic surgical expertise and has a much longer operating time. It is an effective and well-tolerated treatment due to recent advancements in training, surgical techniques, and equipment	High
Nath et al. (2022) [16]	To evaluate the duration of surgery and the need for an oophorectomy between TLH and NDVH and to determine the association between pain after surgery, different intra and postoperative complications, changes in biochemical markers, and length of hospital stay after TLH and NDVH	The patients involved were 50 in the NDVH group and 50 in the TLH group	The mean surgery duration was 54.70 ± 2.862 minutes in the NDVH group and 125 ± 2.373 minutes in the TLH group with a statistically significant difference. With the TLH approach, oophorectomy can be easily performed if adnexal pathology is detected as it offers better visualization with statistically significant results. The mean postoperative pain score (at 12 and 24 hours) was higher in patients in the NDVH group than those in the TLH group. Additionally, the types of operation and intra and postoperative complications were not associated with statistically significant results. TLH patients reported less pain following surgery than NDVH patients. Furthermore, following surgery, a drop in PCV and reduction in Hb was observed in the TLH group with statistically significant results. However, in the NDVH group, a statistically significant decrease in Hb but not PCV was reported. 35 patients in TLH and 43 in the NDVH group out of a total of 50 patients did not show any postoperative complications. The TLH group’s mean hospital stay was 3.54 ± 0.210 days, whereas the NDVH group’s mean hospital stay was 3.66 ± 0.129 days. These differences were not statistically significant	Both techniques reported less blood loss during surgery. The duration of hospitalization was similar for both techniques, and the TLH group had a higher risk of numerous intraoperative complications, such as bladder, ureter, and bowel injuries. In comparison to NDVH, the TLH group demonstrated less pain postoperatively. The appropriate route of surgery should be determined using the standard of care	High

Discussion

The surgical technique used to perform the hysterectomy is determined by the patient’s preoperative morbidity. Numerous past studies have examined the different hysterectomy routes in an attempt to come to an agreement and determine which is the best [17]. Research indicates that minimally invasive surgeries are superior to AH in terms of acceptability, shorter hospital stays, and early return to work; nevertheless, these procedures require specialized skills that can be acquired over time [4,17-19]. For each specific surgical indication, the following factors could influence the hysterectomy technique: accessibility, size, uterine disease, and mobility. The tissue laxity after numerous births, multiparity, and diminished tissue tensile strength provide vaginal surgeons comfort even when there is uterine hypertrophy [4].

According to this systematic review, the most common indication for hysterectomy was uterine fibroids and adenomyotic uterus which is congruous with the studies by Siedhoff et al. [20], Murali et al. [4], and Singh and Soni [5], whereas a previous study conducted by Nagar et al. [21] found adenomyosis followed by fibroid and hyperplasia as the most common indication. According to Desai et al., over 50% of women aged 15 to 49 years self-reported pain or excessive menstrual bleeding as an indication of hysterectomy, followed by fibroids and uterine rupture [22]. Furthermore, in the current review, the NDVH group demonstrated less blood loss and a shorter mean operating time than the TLH group. These findings were found to be similar to previous studies [4,7,21,23-26]. In contrast, Aniuliene et al. performed a retrospective analysis that reported less blood loss during TLH compared to NDVH [27]. Hence, for TLH, using an endoscopic stapler to enhance the skills may reduce time and blood loss.

Intraoperative complications were found to be more common in TLH in comparison to NDVH and the most common complications observed were ureteric and bladder injuries. The most common postoperative complications encountered were urinary tract infections, pyrexia, and ileus. Corresponding with the present systematic review, Shin et al. and Baggish et al. reported 0.6% and 1% of bladder injuries, respectively [28,29]. Moreover, the mean postoperative pain score was less in the TLH technique when compared to the NDVH technique, and therefore, the need for postoperative analgesia requirements was less for TLH in comparison to NDVH. In a prior study, Chattopadhyay et al. found that patients having TLH experienced less pain on the first postoperative day than those undergoing NDVH [30]. These results, however, are not consistent with the study by Tondge et al., which indicated that NDVH experienced less pain than the TLH group [6].

In NDVH, there was a lower conversion to laparotomy (total AH) than in the TLH group, and similar results were reported by a retrospective analysis by Jain et al. that indicated a higher conversion to AH in TLH due to hemorrhage, rectal injury, and bladder injury [31]. Additionally, the mean duration of hospital stay was higher in the TLH group in comparison to NDVH which correlated with the findings of previous studies by Chakraborty et al. [3]. These results contrast with those of the Kansara et al. study, which found no statistically significant difference in the mean duration of hospitalization between TLH and NDVH [15]. Additionally, the duration of hospitalization between three to seven days was observed in other research studies [4,29,32].

Strengths and limitations

This systematic review highlighted the comparison between TLH and NDVH techniques for various outcome measures and discussed the pros and cons associated with both techniques. Moreover, in the systematic review, all included studies were of the highest quality demonstrating the efficacy and effectiveness of both techniques. However, the systematic review described certain limitations which involved, first, a small sample size included in the studies, no reporting of meta-analysis because of the heterogeneous nature in the methodological part of the studies included, and the small number of studies due to the selection criteria imposed. Additionally, studies published in languages other than English were not included, which might have limited the number of relevant studies. Lastly, studies published in journals that are less indexed or published in databases other than those considered were excluded.

## Conclusions

The review concluded that NDVH has an advantage over TLH as a scarless surgery performed in a very short period with minimum blood loss and fewer complications, as well as in terms of cost-effectiveness. Postoperative parameters and satisfaction in TLH may be better than NDVH, as it requires surgical skills and has a significantly longer duration of surgery. Additionally, if adhesions or adnexal masses are present or if salpingo-oophorectomy is indicated, TLH may be the preferred approach. The duration of hospitalization was almost the same in both techniques. As the NDVH technique is easier to learn and can be done by a junior gynecologist as well, it can improve a woman’s well-being and quality of life. In contrast, TLH can only be conducted by a senior surgeon. In conclusion, the decision about the technique should be determined by the pathology and the size of the uterus, the expertise of the surgeon, the technological capabilities of the hospital involved, and the patient’s and surgeon’s preferences.
